# Extraordinary Birth

**Published:** 1853-04

**Authors:** 


*Extraordinauy Birth.*—Mrs. Emma Erchert, of 65 Oxford street, gave birth last week to a female infant with two heads and two necks. One head came into the world nearly four hours before the other. The infant had full vitality two minutes before birth. Dr. Richards, of Bedford-square, acted as accoucheur, and had to use instruments. The body, which was well proportioned, measured 19½ inches in length, and 9½ from shoulder to shoulder, across the back. The mother dreamed, a fortnight previously, that she would give birth to such a monster. Mr. Erchert retains the body, properly preserved.—*Ibid.*

